# Consequences of plateau pika disturbance on plant-soil carbon and nitrogen in alpine meadows

**DOI:** 10.3389/fpls.2024.1362125

**Published:** 2024-02-29

**Authors:** Xue Ting Xu, Yi Mo Wang, Xiao Zheng Wang, Jie Na Li, Jie Li, Ding Yang, Zheng Gang Guo, Xiao Pan Pang

**Affiliations:** ^1^ Key Laboratory of Grassland Livestock Industry Innovation, Ministry of Agriculture and Rural Affairs, State Key Laboratory of Herbage Improvement and Grassland Agro-ecosystems, College of Pastoral Agriculture Science and Technology, Lanzhou University, Lanzhou, China; ^2^ Center for Biological Disaster Prevention and Control, National Forestry and Grassland Administration, Shenyang, China

**Keywords:** grassland ecosystems, bare soil patches, heterogeneity, plant-soil, disturbance intensity

## Abstract

The presence of burrowing mammals can have extensive effects on plants and soils, creating bare soil patches in alpine meadows and potentially altering plant-soil carbon (C) and nitrogen (N). This study focuses on the plateau pika (*Ochotona curzoniae*) to examine the responses of plant-soil C and N to a small burrowing mammal from quadrat scale to plot scale. The density of active burrow entrances in disturbed plots was used as an indicator of the disturbance intensity of plateau pikas. The study found that the below-ground biomass (BGB) and its C and N, as well as soil C and N concentrations were significantly lower in bare soil areas than in vegetated areas and undisturbed plots. This shows that the quadrat scale limited the estimation of the C and N sequestration potential. Therefore, further research on the plot scale found that the disturbance by plateau pika significantly reduced plant biomass and BGB carbon stock. However, plateau pika did not affect soil C and N stocks or ecosystem C and N stocks. These findings suggest the bare soil patches formed by plateau pika caused plant and soil heterogeneity but had a trade-off effect on plant-soil C and N stocks at the plot scale. Nevertheless, moderate disturbance intensity increased the C and N sequestration potential in grassland ecosystems. These results provide a possible way to estimate how disturbance by small burrowing mammals affects C and N cycling in grassland ecosystems while accurately assessing the effects of small burrowing mammal densities on C and N in grassland ecosystems.

## 1 Introduction

Grassland covers approximately 40% of the terrestrial area on Earth (Fang et al., 2018; Ge et al., 2022), and as one of the most important vegetation types on Earth (Zhang et al., 2013), grassland ecosystems are important reservoirs of carbon (C) and nitrogen (N) (Gao et al., 2015; Sitters et al., 2020). The maintenance of C is also a key factor in the sustainability of grassland ecosystems (Huang et al., 2022). Nevertheless, grasslands can be modified by multiple biotic and abiotic factors, and their C and N sequestration potential will be severely influenced (Xia et al., 2009; Eekeren et al., 2010).

Among these factors, burrowing mammal is underappreciated but key functional group worldwide (Davidson et al., 2012), and they can profoundly impact C and N in grassland ecosystems by creating plant and soil disturbances. On the one hand, these burrowing mammals usually have distinct effects on plant growth, which directly changes the absorption of atmospheric carbon dioxide (CO_2_) by plants and its storage in plant biomass (Yurkewycz et al., 2014; Zhang et al., 2014); on the other hand, burrowing and burying effects on the soils of these burrowing mammals changes the organic matter input and decomposition, thereby inevitably influencing the C sequestration potential in the plant-soil systems (Qin et al., 2023). Generally, the C and N retention in the plant-soil systems is assessed by changes in C and N stocks in plants and soils (Deng et al., 2014), and it is often considered an effective agent for estimating the C and N sequestration potential (Reeder and Schuman, 2002; Evans and Burke, 2013).

The plateau pika (*Ochotona curzoniae*) (hereafter pika), one of the dominant species of burrowing mammals in the Qinghai-Tibetan Plateau, is generally considered to be a pest in China due to its accelerated degradation of alpine grasslands (Zhang et al., 2016; Wei et al., 2023). However, some studies have argued that pika is a keystone species for the alpine grassland ecosystem (Smith and Foggin, 1999; Delibes-Mateos et al., 2011). This is because they can modify biotic and abiotic habitat characteristics. For example, pika can serve as food for many predators, and their burrows serve as breeding habitats for small birds and lizards (Smith and Foggin, 1999). Furthermore, pikas break mattic epipedon on the soil surface layer, reducing runoff and increasing water infiltration (Zhang et al., 2014; Wilson and Smith, 2015). Pikas can also excavate soil from deep layers, and excavated soils capture organic matter on the soil surface. This process increases organic matter input and decomposition, releasing nutrients into the soil (Niu et al., 2019). Although increasing attention has been paid to the role of pika in alpine grassland ecosystems, the influence of pika on vegetation and soil is still controversial. This difference could be due to the sampling methods. In fact, the effects of pika can cause heterogeneous microhabitats by creating bare soil patches interspersed in the vegetated soil matrix (Pang et al., 2021). Typically, many studies have focused on the differences between vegetated and bare soil in the presence of pikas (Yi et al., 2016; Zhang et al., 2016; Zhao et al., 2021). Only a few studies have compared soil C and N in vegetated soil areas with and without pikas (Pang and Guo, 2017). These approaches often led to uncertainty in examining the effects of pikas disturbance on soil C and N of alpine grasslands due to ignoring to select areas without pikas as reference (Davidson et al., 2012; Hagenah and Bennett, 2013; Yu et al., 2017b), or neglecting bare soil patches and great heterogeneity (Pang et al., 2020a). A plot-scale method, simultaneously considering the difference between pika presence and absence and the heterogeneity within the presence of pikas, can be a better way to completely estimate the role of pika. Previous studies have shown plant biomass decreased with increasing disturbance intensity (Qin et al., 2021), but soil nutrients showed a slow upward trend followed by a decrease (Pang et al., 2020a, b). However, plant biomass, soil C, and soil N were studied separately, which limited the estimation of C and N sequestration potential under pika disturbance and different disturbance intensities. The changes in the plant-soil systems can better reflect the effects of pikas on grassland ecosystems and deepen the understanding of the role of pika in the C and N cycle of grassland ecosystems. Therefore, more studies are needed to investigate the effects of pika disturbance and disturbance intensity on C and N retention in plant-soil systems at the plot scale.

The effects of pika disturbance on plant-soil C and N in alpine grasslands are examined across multiple sites to improve our knowledge of accurately evaluating C and N sequestration in burrowing mammals. Based on previous studies, we hypothesize that (1) C and N stored in plants are lower in the presence of pikas because of decreased plant biomass, while C and N stored in the soil will be higher due to the capture of organic matter, resulting in a trade-off in plant-soil C and N; (2) higher pika disturbance intensity will decrease plant-soil C and N because of increased bare patches and reduced organic matter input. The results will help to fully elucidate the effects of pika disturbance and disturbance intensity on C and N cycling in grassland ecosystems.

## 2 Materials and methods

### 2.1 Study sites

**Figure 1:**
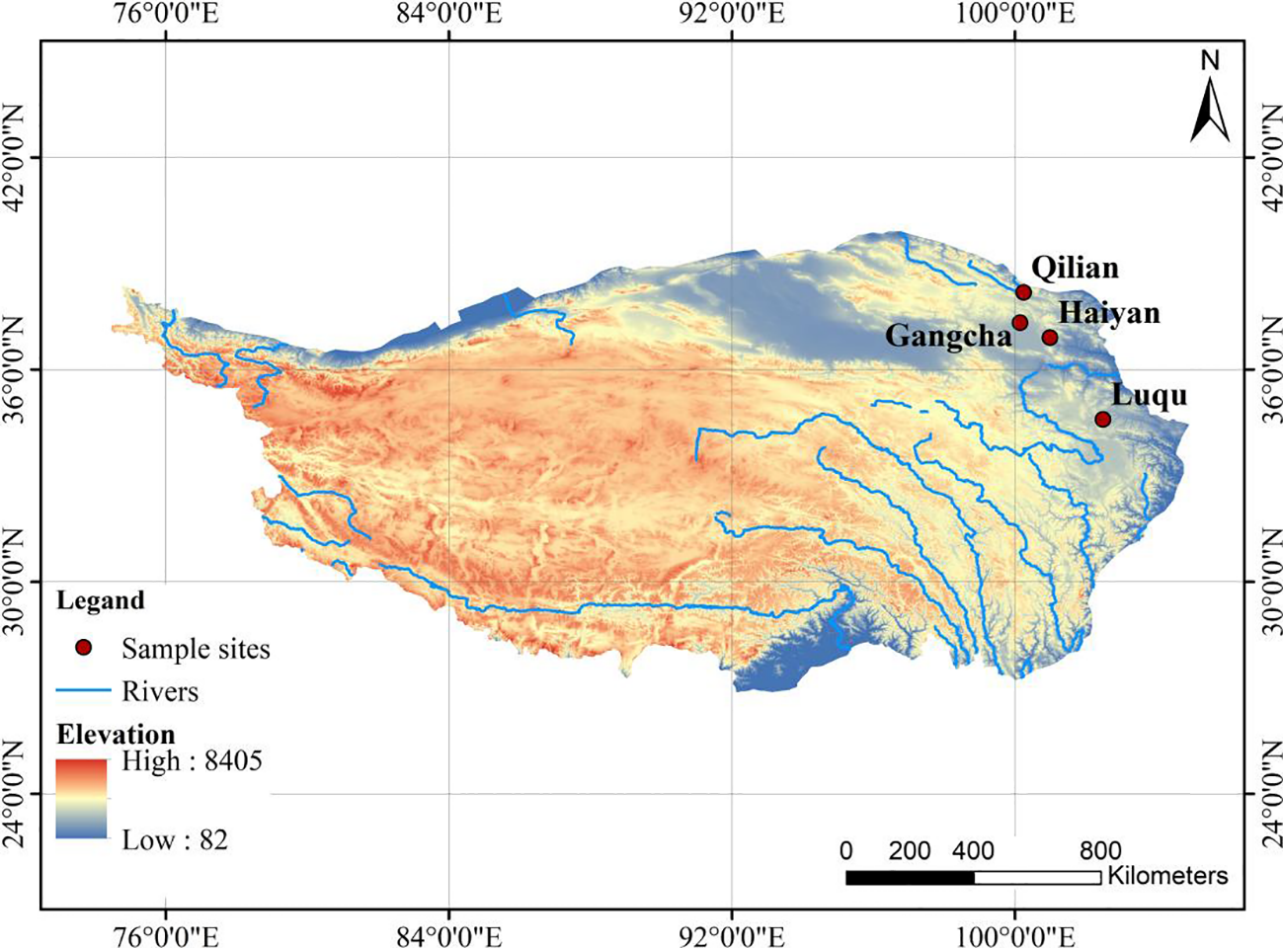
Distribution map of the four study sites.

This study selected four sites on the Tibetan Plateau, located in Gangcha County, Haiyan County, and Qilian County in Qinghai Province and Luqu County in Gansu Province (**Figure 1**), based on the distribution of pikas in existing studies, combined with cross-environmental gradients. These four investigation sites also include, on a large scale, the effects of pika disturbance on plant-soil C and N under different environments on the Tibetan Plateau. The average elevation of these sites varies from 3,169 to 3,550 m, with average annual precipitation ranging from 444 to 691 mm, and average annual temperature ranging from 0.83 to 3.08°C (**Table 1**).

**Table 1:** Elevation, species richness, dominant species, active burrow entrance density, mean annual temperature, and mean annual precipitation recorded from August 2015 to July 2020.

Site	Elevation (m)	MAT (°C)	MAP (mm)	Dominant species	Species richness	ABED (ha^-1^)
Gangcha	3265	0.83	498	*Kobresia humilis*	23.40 ± 2.23 b	1314.72 ± 67.04 a
Haiyan	3270	1.81	444	*K. humilis*	21.53 ± 0.87 b	1456 ± 123.04 a
Luqu	3550	3.08	691	*Kobresia capillifolia*	30.50 ± 0.80 a	664 ± 82.08 b
Qilian	3169	2.36	476	*K. humilis*	19.00 ± 0.58 b	1368 ± 137.92 a

Different lowercase letters show significant differences among the four sites. Values are means ± SE. MAT, mean annual temperature; MAP, mean annual precipitation; ABED, active burrow entrance density.

Alpine meadows are the main grassland type on the Tibetan Plateau (Ma and Zhang, 2022), and pikas are frequently active in alpine meadows. The dominant plant species at the Luqu study site differed from the other three sites (**Table 1**). However, the vegetation types were all categorized as alpine meadows, and their soils were alpine meadow soils (Gong et al., 2007). The soil surface is covered by a root mat composed of plant roots, approximately 7-11 cm thick, covering the soil surface and preventing water infiltration (Pang et al., 2020b). In contrast, constructing burrowing systems by pika destroy the root mat and increase soil water infiltration (Wilson and Smith, 2015; Pang et al., 2021).

### 2.2 Survey design

At each research site, we initially selected 10 disturbed plots affected by pika disturbance, with a distance of 3-5 km between each disturbed plot. Subsequently, we selected adjacent undisturbed plots without pika disturbance for each disturbed plot. The distance between the two-paired disturbed and undisturbed plots was approximately 0.5-1 km. There were no significant differences in grassland type, soil type, topography, microclimate, or vegetation composition between the two-paired disturbed and undisturbed plots to ensure the reliability of the results. Undisturbed plots are considered to be potentially suitable areas for pika (Li et al., 2021). Based on the average area of pika’s home range (Fan et al., 1999), the area of each plot was set as 35 ×35 m. All plots were situated in cold grasslands, where alpine meadows were fenced from mid-April to October to exclude large herbivores.

### 2.3 Sampling and analysis

Field surveys and sampling were conducted in early August 2020, when the annual population of pika was the highest (Fan et al., 1999; Qu et al., 2013), accurately reflecting the effects of pika disturbance on the plant-soil C and N. For plots with pikas, the active burrow entrance density (ABED) was used as a proxy for the intensity of the disturbance (Sun et al., 2015). The disturbed plot consisted of vegetated and bare soil surfaces. First, the area of bare soil in disturbed plots was determined by the sum of the areas of bare patches using the split method (Wei and Guo, 2022). Next, five vegetated quadrats (1×1 m) approximately 8 m apart were placed along a W-shape on the vegetated surface of each disturbed plot, and the paired bare soil quadrats were selected for each vegetated quadrat. The distance between each paired vegetated quadrat and bare soil quadrat should not be too far from each other, which was about 1 m. For each undisturbed plot, five vegetated quadrats were placed similarly. Therefore, there are 15 quadrats for each paired undisturbed-disturbed plot, i.e., five vegetated quadrats and five bare soil quadrats within each disturbed plot, and five quadrats within each undisturbed plot.

In this study, all the bare soil patches in the disturbed plots were new bare soil patches with no vegetation cover, so above-ground biomass (AGB) and its C and N at the plot scale, defined as AGB and its C and N measured on the surface of vegetation adjusted for the lack of AGB in bare soil patches, may properly assess the response of AGB and its C and N to the disturbance by small burrowing mammal. For each quadrat, all plants were clipped at ground level, and then a root auger (10 cm in diameter) was used to obtain the plant roots at a depth of 20 cm. The clipped plants were oven-dried in the laboratory at 65°C (48 h) and weighed to calculate AGB. Then, the plant roots were carefully washed with water over a 0.5 mm sieve, dried, and weighed to estimate the below-ground biomass (BGB). Total biomass is the sum of AGB and BGB. Five soil cores (3.5 cm in diameter, 0-20 cm depth) were randomly collected from each quadrat after plant samples were collected, mixed, and passed a 2 mm sieve to remove stones and roots. Meanwhile, soil profiles of 20 cm depth were dug using a stainless-steel cutting ring (the volume was 100 cm^3^) to collect soil cores to determine soil bulk density. Plant and soil C and N concentrations were analyzed using dry combustion.

### 2.4 Calculation of plant, soil, and ecosystem C and N stocks

#### 2.4.1 The plant C and N stocks (including AGB and BGB carbon and nitrogen stocks) of disturbed and undisturbed plots were calculated as follows:


(1) $$C/N_{A-stock-dist}\;=\;C/N_{stock-VA}\;\times \;\left(1\;-\;BA\right)$$

where C/N_A-stock-dist_ is AGB carbon or nitrogen stock of disturbed plots (g m^−2^); C/N_stock-VA_ is AGB carbon or nitrogen stock of vegetated areas within disturbed plots (g m^−2^); BA is the area (%) of bare soil.


(2) $$C/N_{B-stock-dist}\;=\;C/N_{stock-BB}\;\times \;BA\;+\;C/N_{stock-VB}\;\times \;VA$$

where C/N_B-stock-dist_ is BGB carbon or nitrogen stock of disturbed plots (g m^−2^); C/N_stock-BB_ is BGB carbon or nitrogen stock of bare soil areas (g m^−2^); C/N_stock-VB_ is BGB carbon or nitrogen stock of vegetated areas within disturbed plots (g m^−2^); BA and VA are area (%) of bare soil and vegetated soil within disturbed plots.


(3) $$C/N_{P-stock-undist}\;=\;B_{undist}\;\times \;C/N_{UP}$$

where C/N_P-stock-undist_ is plant C or N stock of undisturbed plots (g m^−2^); B_undist_ is plant biomass of undisturbed plots (g m^−2^); C/N_UP_ is plant C or N concentration of undisturbed plots (%).


(4) $$B_{dist}=B_{BP}\times BA\times B_{VP}\times VA$$

where B_dist_ is the plant biomass of disturbed plots (g m-2); B_BP_ is the plant biomass of bare soil areas (g m^−2^); B_VP_ is the plant biomass of vegetated areas within disturbed plots (g m^−2^); BA and VA are area (%) of bare soil and vegetated soil within disturbed plots.

#### 2.4.2 The soil C and N stocks of disturbed and undisturbed plots were calculated as follows:


(5) $$C/N_{s-stock-dist}\;=\;C/N_{stock-BS}\;\times \;BA\;+\;C/N_{stock-VS}\;\times \;VA$$

where C/N_s-stock-dist_ is soil C or N stock of disturbed plots (g m^−2^); C/N_stock-BS_ is the C or N stock of bare soil (g m^−2^); C/N_stock-VS_ is the C or N stock of vegetated soil within disturbed plots (g m^−2^); BA and VA are area (%) of bare soil and vegetated soil within disturbed plots. The area of bare soil in undisturbed plots was 0, and the area of vegetated soil in undisturbed plots was 100% because only bare soil areas resulting from pika activities were considered in this study.


(6) $$C/N_{s-stock-undist}\;=\;\left[C/N_{US}\;\times \;BD_{US}\;\times \;T\;\times \;\left(1\;-\;\delta_{US}\right)\;\times \;0.01\right]\;\times \;100\%$$

where C/N_s-stock-undist_ is the C or N stock of undisturbed soil (g m^−2^) and C/N_US_, BD_US_, and δ_US_ are soil C or N concentration (%), soil bulk density (g cm^−3^), and soil fraction of gravel larger than 2 mm of undisturbed soil respectively.

Stocks of C and N in grassland ecosystems were estimated by summing the plant and soil C and N stocks.

### 2.5 Data analysis

All statistical analyses were performed using R 4.0.3 (R Foundation for Statistical Computing, Vienna, Austria). The effects of pika disturbance on plant biomass, plant C and N concentrations and stocks, and soil C and N concentrations and stocks at four sites were assessed at two scales using a linear mixed model (LMM) with the “lme4” package, where the presence/absence of pikas disturbance and the four sites were fixed factors, and paired designs were random factors. Tukey’s tests were then performed for *post hoc* comparisons using the “emmeans” package. A regression analysis was constructed with ABED as a fixed factor to elucidate the changes in C and N stocks in grassland ecosystems to the disturbance caused by pika. The regression curves of ABED and C and N stocks in grassland ecosystems at the plot scale were obtained using a linear model (LM).

## 3 Results

### 3.1 The responses of plant biomass and its C and N to pika disturbance at the quadrat scale

**Figure 2:**
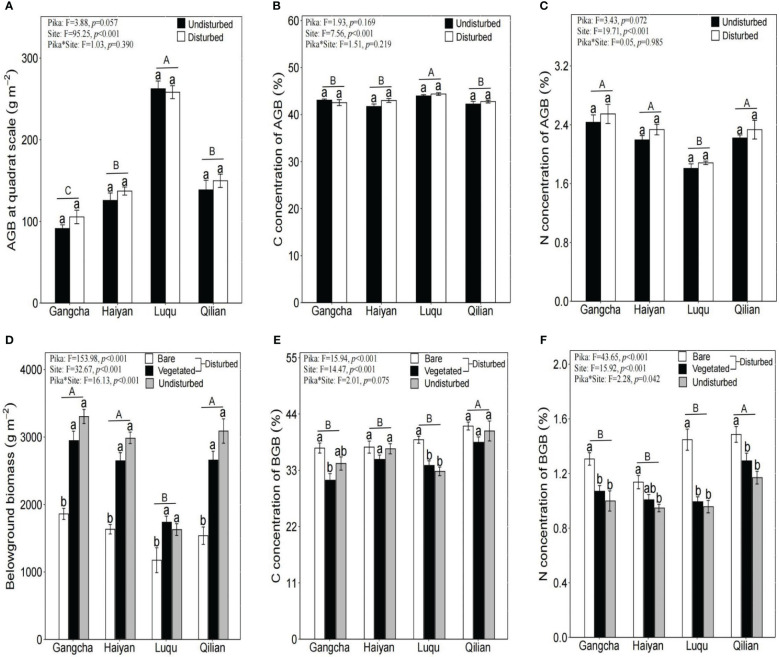
Plant biomass (**A:** AGB, **D:** BGB) and its C and N (**B:** C concentration of AGB, **C:** N concentration of AGB, **E:** C concentration of BGB, **F:** N concentration of BGB) at the quadrat scale (mean ± SE). The statistics were obtained from the paired T-test for each site. Different lowercase letters indicate significance between disturbed and undisturbed plots; different uppercase letters indicate significance between sites when the presence and absence of pikas are combined for each site, *P<* 0.05.

Pika disturbance did not affect AGB or its C and N concentrations at the quadrat scale. BGB was significantly lower in bare soil areas than in vegetated areas and undisturbed plots (**Figure 2**). Nevertheless, the N concentration of BGB was significantly higher in bare soil areas, and its C concentration was not significantly different between bare soil areas, vegetated areas, and undisturbed plots (**Figure 2**).

### 3.2 The responses of soil C and N to pikas disturbance at the quadrat scale

**Figure 3:**
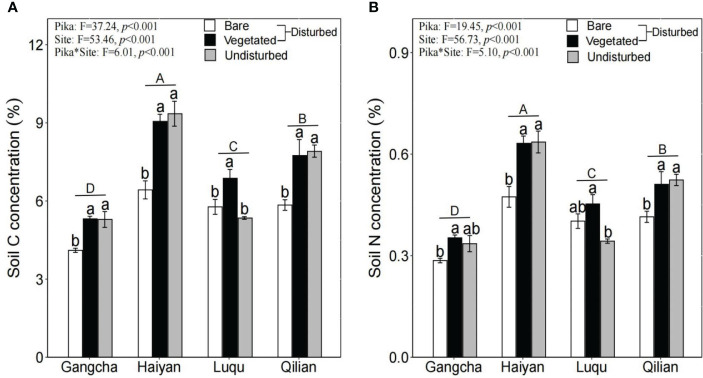
Soil C **(A)** and N **(B)** concentrations in bare soil areas, vegetated areas within disturbed plots, and undisturbed plots at each site (mean ± SE). The statistics were obtained from the paired T-test for each site. Different lowercase letters indicate significance between disturbed and undisturbed plots; different uppercase letters indicate significance between sites when the presence and absence of pikas are combined for each site, *P<* 0.05.

The study found that changes in soil C and soil N concentrations were similar. Specifically, soil C and N concentrations were significantly lower in bare soil areas than in vegetated areas and undisturbed plots (**Figure 3**).

### 3.3 Effects of pika disturbance on plant biomass at the plot scale

**Figure 4:**
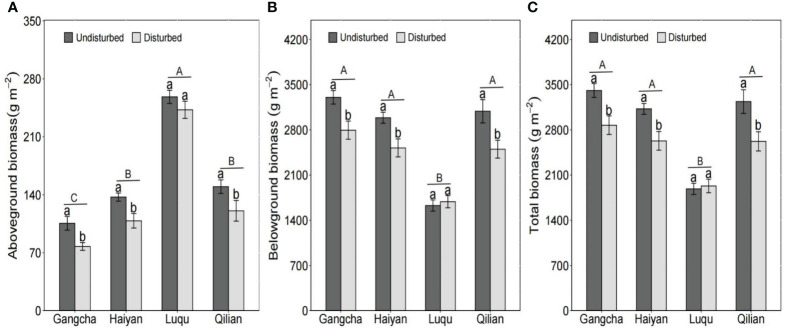
Response of above-ground biomass **(A)**, below-ground biomass **(B)**, and total biomass **(C)** to the disturbance by pikas at the plot scale (mean ± SE). The statistics were obtained from the LMMs, using paired plots at each site as random factors. Different lowercase letters indicate significance between disturbed and undisturbed plots; different uppercase letters indicate significance between sites when the presence and absence of pikas are combined for each site, *P<* 0.05.

According to the study investigations, plant biomass response to disturbance by pikas was similar at all sites except for Luqu. Disturbance by pikas significantly reduced plant biomass (**Figure 4**). The total biomass in disturbed plots of Gangcha, Haiyan, and Qilian were 18.69%, 18.86%, and 23.52% lower than those in undisturbed plots, respectively. In addition, total biomass was lower at Luqu than at the other three sites (**Figure 4**). This result is similar to studies at the quadrat scale.

### 3.4 Response of plant-soil C and N to disturbance by pikas at the plot scale

**Figure 5:**
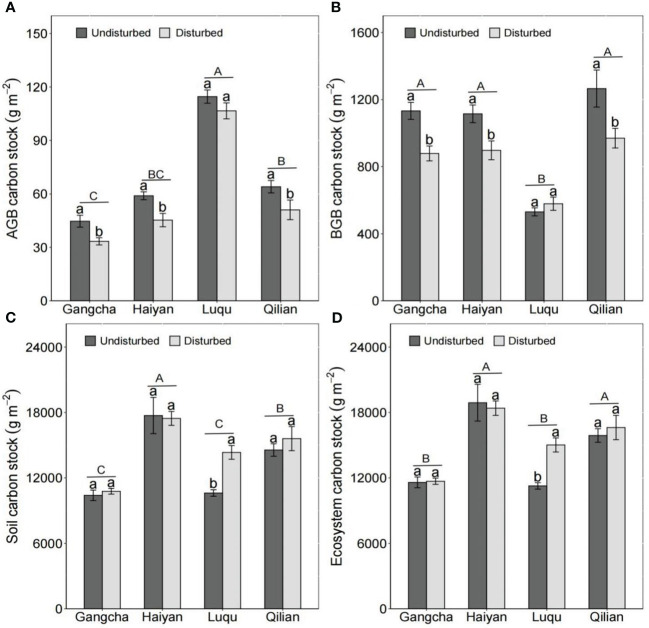
AGB carbon stock **(A)**, BGB carbon stock **(B)**, soil carbon stock **(C)**, and ecosystem carbon stock **(D)** in plots disturbed and undisturbed by pikas at each site (mean ± SE). The statistics were obtained from the paired T-test for each site. Different lowercase letters indicate significance between disturbed and undisturbed plots; different uppercase letters indicate significance between sites when the presence and absence of pikas are combined for each site, *P<* 0.05.

**Figure 6:**
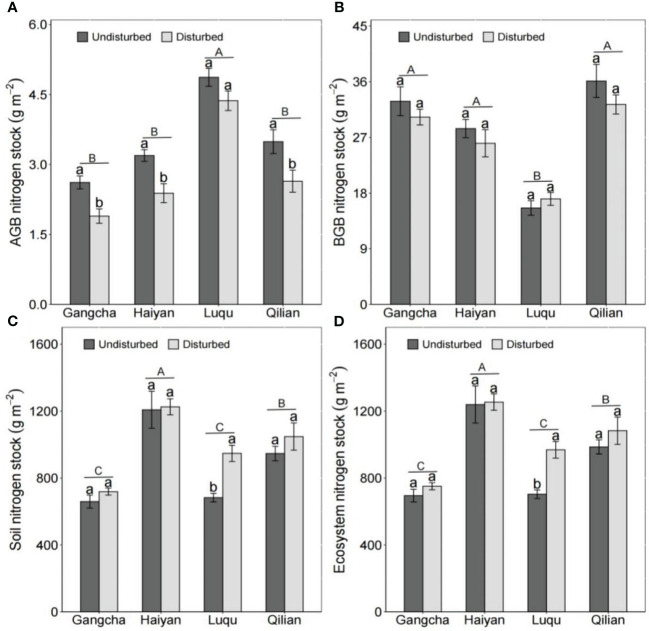
AGB nitrogen stock **(A)**, BGB nitrogen stock **(B)**, soil nitrogen stock **(C)**, and ecosystem nitrogen stock **(D)** in plots disturbed and undisturbed by pikas at each site (mean ± SE). The statistics were obtained from the paired T-test for each site. Different lowercase letters indicate significance between disturbed and undisturbed plots; different uppercase letters indicate significance between sites when the presence and absence of pikas are combined for each site, *P<* 0.05.

The disturbance caused by pika reduced AGB carbon and nitrogen stocks and BGB carbon stocks but had no effect on C and N in grassland ecosystems (**Figures 5**, **6**). The studies in Gangcha, Haiyan, and Qilian are consistent with these results. The AGB carbon and AGB nitrogen stocks in disturbed plots of Gangcha, Haiyan, and Qilian were 33.86% and 38.17%, 30.15% and 33.86%, and 25.42% and 32.05% lower than those in undisturbed plots, respectively. The BGB carbon stocks in disturbed plots of Gangcha, Haiyan, and Qilian were 28.91%, 24.33%, and 30.53% lower than those in undisturbed plots, respectively.

However, the effects of disturbance by pikas on C and N stocks were site-specific. Disturbance by pikas had no impact on plant C and N stocks, but significantly increased soil C and N stocks at Luqu (**Figures 5**, **6**). Thus, pika disturbance increased ecosystem C and N stocks at Luqu. The C and N stocks in grassland ecosystems were 33.30% and 37.68% higher in disturbed plots than in undisturbed plots, respectively.

When combining the presence or absence of pikas, Luqu had higher AGB carbon and nitrogen stocks but lower BGB carbon and nitrogen stocks than Gangcha, Haiyan, and Qilian; and Haiyan had higher ecosystem C and N stocks (**Figures 5**, **6**).

### 3.5 Effects of pika disturbance intensity on plant-soil C and N in alpine meadow ecosystems

**Figure 7:**
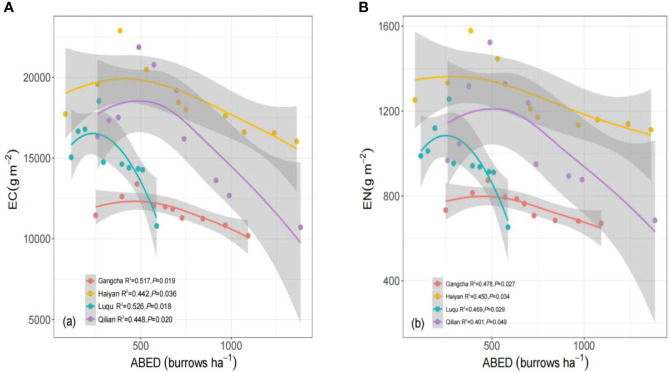
Ecosystem carbon **(A)** and nitrogen **(B)** stocks under different pika disturbance intensities based on linear models (lm) for each site. An adjusted local smoothed regression line (color) with its 95% confidence interval (gray) was used to determine the relationship between the disturbance intensity of pikas and the indicators above. EC, ecosystem carbon stocks; EN, ecosystem nitrogen stocks; ABED, active burrow entrance densities.

The C and N stocks in grassland ecosystems increased with increased disturbance intensity in Gangcha, Haiyan, Luqu, and Qilian (**Figures 7A, B**). The ecosystem C and N stocks showed a fast or a slow decreasing trend when the ABED reached a certain value (about 268-489 ha^-1^).

## 4 Discussion

Pikas are a key component of alpine meadow ecosystems (Wei et al., 2019). The presence of pikas can cause extensive disturbance to plants and soils, potentially altering plant-soil C and N and affecting C and N cycling in grassland ecosystems (Zhao et al., 2022).

### 4.1 Effects of plateau pika disturbance on plant-soil C and N at the quadrat scale

This study shows that pika disturbance did not affect AGB and its C and N concentrations (**Figure 2**). Further research found that BGB, soil C, and soil N concentrations were significantly lower in bare soil areas than in vegetated areas and undisturbed plots (**Figures 2**, **3**). The trend of soil C and N is consistent because N in the soil is mainly bound in the form of organic N compounds (Kelley and Stevenson, 1995). However, the results for soil C and soil N contradicted the first hypothesis. Soil nutrients were also contrary to the study of Yu et al. (2017a), possibly due to inconsistencies in sampling methods. They did not differentiate between bare soil and vegetated areas when collecting soil samples from disturbed areas. The impact of pikas on alpine grassland vegetation and soil is not uniform within the home range of pika families (Wei et al., 2019). Moreover, we studied new bare patches with no vegetation covering the surface of the bare patches. Therefore, there may be three mechanisms for lower soil C and N stocks in bare soil areas: first, reducing vegetation and roots in bare soil areas can decrease the input of soil organic matter (Yu et al., 2017a); second, the digging behavior of pikas moves deep soil with lower organic matter content to the topsoil (Clark et al., 2016); third, the mineralization of soil organic matter in bare soil areas is high (Yu et al., 2017b). However, the N concentration of BGB was significantly higher in bare soil areas (**Figure 2**). Patterns of C and N nutrient partitioning in above- and below-ground plant organs are among the most important strategies for plant communities to adapt to external changes (Liu et al., 2021). Therefore, the increase in the N concentration of BGB in bare soil areas may be caused by the above-ground vegetation in bare soil areas being heavily foraged and clipped by pikas, resulting in a smaller supply of N to the above-ground vegetation. Plant roots also increased the uptake of N to promote growth. Additionally, effective N increased in bare soil areas (Pang et al., 2020b), and more N became available for uptake and utilization by the roots. These findings suggest that plants and soils in bare soil areas are highly heterogeneous, contributing to the understanding of the role of the pika on C and N cycling in bare patches. Therefore, further research on the relationship between pika disturbance and C and N at the plot scale is required.

### 4.2 Effects of plateau pika disturbance on plant-soil C and N at the plot scale

At the plot scale, pika disturbance significantly reduced plant biomass, AGB carbon and nitrogen stocks, and BGB carbon stocks (**Figures 4**–**6**). The C and N stored in plants decreased as plant biomass decreased. The effects of pikas on plant C and N were consistent with the first hypothesis. However, there were no differences in BGB carbon, soil C, soil N, ecosystem C, and ecosystem N stocks between disturbed and undisturbed plots (**Figures 5**, **6**). The C and N stored in grassland ecosystems are mainly determined by C and N in grassland soils (Ni, 2002), so pika disturbance maintained the C and N balance of grassland ecosystems. Thus, pika disturbance has a trade-off effect on plant-soil C and N stocks at the plot scale, showing that small mammals interfere differently than large mammals. For example, the trampling of and grazing on grasslands by livestock grazing are more severe, resulting in large-scale losses of grassland vegetation and sources of soil C and N (Pringle et al., 2014; Zhou et al., 2017). However, the effects of disturbance by pikas on plant biomass and plant-soil C and N stocks were site specific. In the alpine meadows of Luqu, disturbance by pikas had no impacts on plant biomass and AGB carbon and nitrogen stocks but significantly increased soil C and N stocks (**Figures 4**–**6**). Possibly due to higher precipitation and temperature, the dominant plant species at the Luqu study site was *K. capillifolia* with dense above-ground vegetation (**Figure 4** and **Table 1**). Disturbance by pikas can increase the deposition rate of uneaten food and tall plant clippings, thereby increasing the input of organic matter (Liu et al., 2013; Zhang et al., 2016). Moreover, the abundant vegetation was clipped and buried in burrows by pikas, thus storing part of the soil organic matter (Yurkewycz et al., 2014; Clark et al., 2016). Soil C and N are also related to pika density and excretion of feces and urine, contributing to the input of soil organic matter (Yu et al., 2017a). Conversely, higher soil C and N sustain plant growth and development. The survey also found that when combining the presence and absence of pikas, AGB and its C and N stocks were higher but BGB, BGB carbon and nitrogen stocks, and total biomass were lower at Luqu (**Figures 4**– **6**). The average annual precipitation was approximately 691 mm, and the average annual temperature was 3.08°C at Luqu. The relatively high precipitation and temperature favor the growth of above-ground vegetation (Holub, 2002; Ma et al., 2010), but can make the plant roots undeveloped (Ibrahim et al., 1997; Bakker et al., 2006; Qaderi et al., 2010; Xu et al., 2016). The study also found that C and N stored in soil and ecosystem were higher at Haiyan (**Figures 5**, **6**), which could be attributed to the lower temperatures, slowing down the decomposition of soil organic C, thus storing soil organic C.

### 4.3 Relationship between disturbance intensity and C and N in grassland ecosystems at the plot scale

C and N stocks in grassland ecosystems showed hump-shaped changes with increasing disturbance intensity at all four sites when analyzing the relationship between the disturbance intensity (measured by ABED) and ecosystem C and N stocks. Ecosystem C and N stocks decreased with increasing disturbance intensity when the disturbance intensity exceeded the threshold. This result partially supports the second hypothesis, which may be explained by reducing soil C and N sources at high disturbance intensity (Sun et al., 2015; Pang and Guo, 2017). This result may also be explained by the increase in soil permeability, resulting in the loss of soil C and N (Wilson and Smith, 2015). These findings from the study detail the general pattern of pika disturbance on plant-soil C and N stocks at each site, deepening the understanding of the relationship between small burrowing mammal disturbance and grassland ecosystems on the Tibetan Plateau. Nevertheless, the effects of bare patches of different ages on C and N in grassland ecosystems could not be fully evaluated because the disturbed plots in this study were all new bare patches, and no restored bare patches were considered.

## 5 Conclusions

This study investigated the relationship between pika disturbance and plant-soil C and N at two scales. The findings suggest that the bare soil patches formed by pikas caused plant and soil heterogeneity but had a trade-off effect on plant-soil C and N stocks at the plot scale. Nevertheless, moderate disturbance intensity increased the C and N sequestration potential in the grassland ecosystems. These findings in this study provide possible ways to estimate how disturbance by small burrowing mammals affects C and N cycling in grassland ecosystems and to accurately assess the effects of small burrowing mammal densities on C and N in grassland ecosystems.

## Data availability statement

The raw data supporting the conclusions of this article will be made available by the authors, without undue reservation.

## Author contributions

XX: Data curation, Formal Analysis, Writing – original draft, Writing – review & editing. YW: Data curation, Formal Analysis, Writing – original draft, Writing – review & editing. XW: Data curation, Writing – review & editing. JNL: Data curation, Writing – review & editing. JL: Investigation, Writing – review & editing. DY: Investigation, Writing – review & editing. ZG: Investigation, Writing – review & editing. XP: Data curation, Formal Analysis, Investigation, Writing – review & editing, Funding acquisition.
